# The Role of *SHI/STY/SRS* Genes in Organ Growth and Carpel Development Is Conserved in the Distant Eudicot Species *Arabidopsis thaliana* and *Nicotiana benthamiana*

**DOI:** 10.3389/fpls.2017.00814

**Published:** 2017-05-23

**Authors:** Africa Gomariz-Fernández, Verónica Sánchez-Gerschon, Chloé Fourquin, Cristina Ferrándiz

**Affiliations:** Instituto de Biología Molecular y Celular de Plantas, Consejo Superior de Investigaciones Científicas–Universidad Politécnica de ValenciaValencia, Spain

**Keywords:** carpel evolution, *Eschscholzia californica*, gynoecium, *Nicotiana benthamiana*, style and stigma, virus-induced gene silencing (VIGS), SHI STY SRS factors

## Abstract

Carpels are a distinctive feature of angiosperms, the ovule-bearing female reproductive organs that endow them with multiple selective advantages likely linked to the evolutionary success of flowering plants. Gene regulatory networks directing the development of carpel specialized tissues and patterning have been proposed based on genetic and molecular studies carried out in *Arabidopsis thaliana*. However, studies on the conservation/diversification of the elements and the topology of this network are still scarce. In this work, we have studied the functional conservation of transcription factors belonging to the SHI/STY/SRS family in two distant species within the eudicots, *Eschscholzia californica* and *Nicotiana benthamiana*. We have found that the expression patterns of *EcSRS-L* and *NbSRS-L* genes during flower development are similar to each other and to those reported for Arabidopsis *SHI/STY/SRS* genes. We have also characterized the phenotypic effects of *NbSRS-L* gene inactivation and overexpression in Nicotiana. Our results support the widely conserved role of *SHI/STY/SRS* genes at the top of the regulatory network directing style and stigma development, specialized tissues specific to the angiosperm carpels, at least within core eudicots, providing new insights on the possible evolutionary origin of the carpels.

## Introduction

Organ development is directed by gene regulatory networks (GRNs) that control the temporal and spatial expression of downstream effectors responsible for creating morphogenetic outputs. GRNs are composed mainly of transcription factors and other regulators of gene expression such as microRNAs or chromatin-modifying elements, and signaling molecules such as mobile peptides or hormones, that interact extensively at different levels to generate morphogenetic patterns (Davidson and Levine, 2008). Over the last few decades, a wealth of genetic and molecular studies has uncovered major players that participate in organ formation and patterning in plants, and more recently, system biology approaches have complemented these studies to generate global and more detailed pictures of the regulatory networks that underlie organ development. Most of these studies have been carried out in the model species *Arabidopsis thaliana*, where, for example, meristem formation, root development, or floral organ specification are increasingly understood from this global perspective (Alvarez-Buylla et al., 2007; De Lucas and Brady, 2013; O’Maoileidigh et al., 2014; Tian and Jiao, 2015; Davila-Velderrain et al., 2016). In addition, the rapidly increasing toolkit of genetic, molecular and functional resources across the plant kingdom is also expanding this knowledge to many other species and boosting evo-devo approaches to understand the evolution of plant form (Vialette-Guiraud et al., 2016).

Angiosperms are the largest and most diverse group of land plants. Carpels are the ovule-bearing structures of angiosperms, representing a major evolutionary innovation for this group that was probably key for their success. The carpels provide a confined casing to protect the ovules in their development. In the flower they may occur as single carpels, multiple unfused carpels or a syncarpic structure resulting from multiple fused carpels, where each individual structure is termed pistil. While morphological diversity of pistils is huge across angiosperms, they mostly share a basic organization plan. Apically, specialized cells form the stigma, which receives, discriminates and helps to germinate the pollen grains. The stigma is connected to the ovary through the style, generally a tube-like structure containing transmitting tissues that serve to grow and direct the pollen tubes toward the ovules. The ovary occupies a basal position, housing the ovules and, upon fertilization, it becomes a fruit, which may or may not incorporate additional parts of the flower, and serves to protect the developing seeds and later to facilitate seed dispersal (Ferrandiz et al., 2010). GRNs directing pistil patterning have been studied in Arabidopsis. Several genetic and hormonal factors required for the specification of carpel identity or the development of the specialized pistil tissues have been identified in the last few years, as well as some of their interactions and regulatory hierarchies. From these studies, GRNs directing the different functional modules in the Arabidopsis carpels have been proposed, and, although we are still far from completing an integrated network that provides a comprehensive view of spatial–temporal pistil morphogenesis, we increasingly understand how the basic blocks that compose a functional pistil are formed (Ferrandiz et al., 2010; Reyes-Olalde et al., 2013; Chávez Montes et al., 2015; Ballester and Ferrandiz, 2016; Marsch-Martinez and de Folter, 2016).

The evolutionary importance of carpels has also prompted many different labs to explore questions mainly focused on the evolutionary origin of carpels and the conservation of the genetic functions that specify carpel identity (Bowman et al., 1989; Bradley et al., 1993; Davies et al., 1999; Pan et al., 2010; Yellina et al., 2010; Dreni et al., 2011; Fourquin and Ferrandiz, 2012). However, only more recently these studies are being extended to other components of the emerging GRN proposed to direct pistil patterning. The increasing availability of plant genome sequences has allowed to reconstruct phylogenies for many of the gene families involved in carpel development with good taxonomic sampling, and thus to propose hypotheses on the possible evolution of the pistil GRNs (Pabon-Mora et al., 2014; Pfannebecker et al., 2017a,b). However, it is necessary to complement these works by carrying out functional studies in different taxa, which are still scarce. In this context, it is especially interesting to explore the functional conservation of the elements that drive style and stigma specification, since these tissues are only found across angiosperms and intimately linked to the evolutionary origin of carpels.

In Arabidopsis, it has been shown that correct auxin signaling is essential to establish apical-basal polarity in the Arabidopsis pistil, for correct development of the style and stigma and to ensure apical closure (Sundberg and Ostergaard, 2009; Larsson et al., 2013; Larsson et al., 2014; Moubayidin and Ostergaard, 2014). Two families of transcription factors are essential for style and stigma formation, and, at least in part, they exert these functions by regulating auxin synthesis, transport and response. The four *NGATHA (NGA)* genes belong to the RAV clade of the B3-domain transcription factor family and act redundantly to specify style and stigma identity. The Arabidopsis *nga* quadruple mutants completely fail to form these apical tissues and are female sterile, but develop fairly normal ovaries (Alvarez et al., 2009; Trigueros et al., 2009). The SHI/STY/SRS family of zinc-finger transcription factors, named after the members of this family in Arabidopsis SHORT INTERNODES (SHI), STYLISH (STY), and SHI RELATED SEQUENCE (SRS), play similar roles in pistil development, and multiple combinations of mutants in four or more members of the family cause almost identical phenotypes to those of quadruple *nga* mutants (Kuusk et al., 2002, 2006). NGA and SHI/STY/SRS factors share similar patterns of expression and common targets, and appear to work as master regulators of the GRN directing style and stigma development. In fact, simultaneous overexpression of *NGA3* and *STY1* in Arabidopsis is sufficient to direct ectopic style tissue formation to the whole surface of the ovary (Kuusk et al., 2002, 2006; Sohlberg et al., 2006; Trigueros et al., 2009; Staldal et al., 2012; Martinez-Fernandez et al., 2014). The role of NGA orthologs in pistil development is strongly conserved in distant species such as the basal eudicot *Eschscholzia californica* or the asterid core eudicot *Nicotiana benthamiana*, where *NGA* inactivation leads to the absence of style and stigma differentiation (Fourquin and Ferrandiz, 2014). Several reports show that the connection of *SHI/STY/SRS* genes to auxin signaling is also conserved across land plants, as well as a general role in controlling plant architecture and possibly other hormone pathways (Eklund et al., 2010a,b; Zawaski et al., 2011; Islam et al., 2013; Youssef et al., 2017). However, the role of *SHI/STY/SRS* genes in pistil development has not been explored in detail outside Brassicaceae, although it has been described that barley mutants in the *Lks2* gene, a member of the SHI/STY/SRS family, have short awns and defective stigmas (Yuo et al., 2012).

In this work, we have studied whether members of the SHI/STY/SRS family have conserved roles in style and stigma development in *E. californica* and *N. benthamiana*. This study supports that, as shown for *NGA* genes, SHI/STY/SRS factors are essential for the development of the apical tissues of pistils at least in the core eudicots and that their downstream effectors leading to apical-basal patterning in the carpels are also likely conserved.

## Results

### Identification of *SHI/STY/SRS* Genes in *E. californica* and *N. benthamiana*

The *SHI/STY/SRS* gene family in Arabidopsis comprises 10 members. Published phylogenies show that they belong to a plant-specific family, where homologs can be found already in the moss *Physcomitrella patens* or the lycophyte *Selaginella moellendorffii* (Eklund et al., 2010b; Pfannebecker et al., 2017a). SHI/STY/SRS factors share two highly conserved domains: a C3HC3H RING zinc finger domain and a IGGH motif that appears to be specific to this family (Fridborg et al., 2001), but outside these conserved regions the protein sequences are highly divergent (**Supplementary Table S1**).

**FIGURE 1:**
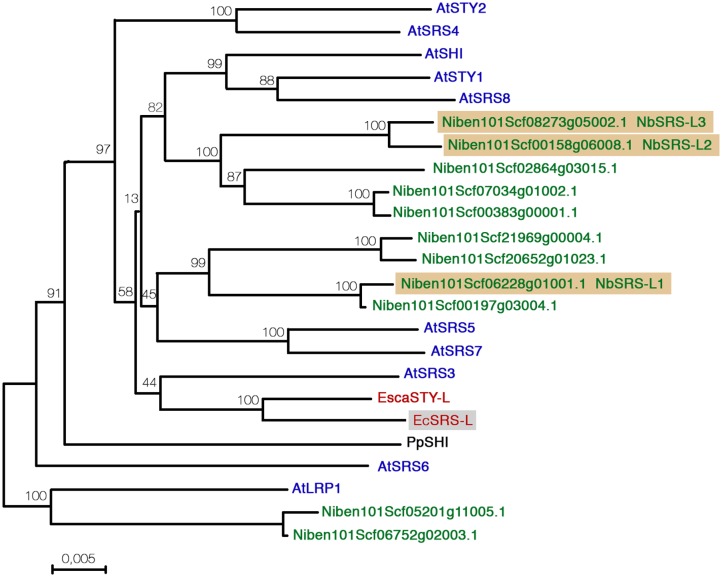
**Distance analysis between *Nicotiana benthamiana*, *Eschscholzia californica*, and *Arabidopsis thaliana* SHI/STY/SRS factors.** Sequences were aligned with Muscle and the phylogenetic tree was generated using MEGA7 software using the Neighbor-Joining method. The optimal tree is shown. The percentage of replicate trees in which the associated protein sequences clustered together in the bootstrap test (2000 replicates) are shown next to the branches.

To search for *SHI/STY/SRS* homologs in *E. californica*, we designed degenerate primers based on the sequence the RING-like and IGGH conserved domains of *SHI/STY/SRS* genes from other species. Only one putative *SHI/STY/SRS* gene, named *EcSRS-L*, was amplified from cDNA of *E. californica* flowers. The complete coding sequence of *EcSRS-L* was subsequently amplified by TAIL PCR and by the use of an adapted oligo-dT primer. The predicted EcSRS-L protein sequence possessed the typical RING domain and IGGH motif, but was different from the EscaSTY-L protein sequence recently published (Pfannebecker et al., 2017a), that we were not able to amplify with this strategy, probably because it contains a variant of the IGGH domain that does not align with our degenerate primers (**Supplementary Figure S1**). Eleven SRS-related sequences were identified by searching the most recent draft of *N. benthamiana* genome (**Figure 1**) (Bombarely et al., 2012), all of them coding for predicted proteins that contained the RING and IGGH domains.

Using the identified *E. californica* and *N. benthamiana* predicted protein homologs and the Arabidopsis protein sequences of the SHI/STY/SRS family members we performed comparative sequence analyses, using the Neighbor joining algorithm built into the Mega7 software (**Figure 1**). The resulting tree had an overall topology similar to that of other previously published, although some differences were found mainly affecting less-supported clades and probably due to the use of different tree reconstruction methods and datasets. The clade comprising AtSTY1, AtSHI, and AtSRS8 was related to one formed by five Nicotiana predicted proteins. Four additional Nicotiana proteins grouped in a well-supported clade related to AtSRS5 and AtSRS7, while both EcSRS-L and EscaSTY-L grouped in the same clade as AtSRS3. Finally, two Nicotiana predicted proteins clustered as an outgroup with AtLRP. Low support of some branches did not allow to unequivocally establish direct relationships of some of the *N. benthamiana* and *E. californica* factors with the Arabidopsis SHI/STY/SRS proteins, but the tree strongly suggested that the duplication events that resulted in the high number of homologs found in Arabidopsis and Nicotiana were independent, and also that in Nicotiana, a recent allotetraploid, two copies of each gene were usually present.

Functional studies in Arabidopsis have shown that members of the family have redundant functions and that the degree of this redundancy does not depend strongly on how similar are their sequences. Thus, *sty1-1* is the only single mutant that shows an abnormal phenotype in gynoecium development, but combinations with mutant alleles in either *LRP*, *SRS5*, *STY2*, or *SHI* genes, which belong to different subclades in the family and have, respectively, a similarity score of 30, 34, 42, and 54% with STY1, similarly enhance the *sty1-1* defects in carpel development (Kuusk et al., 2002, 2006). For this reason, and since none of the *NbSTY/SHI/SRS* genes showed a clear orthology relationship with *AtSTY1*, we decided to focus for this study on three *N. benthamiana* genes: Niben101Scf06228g01001.1, a member of a sister clade to the one formed by the Arabidopsis *AtSRS5* and *AtSRS7* genes, and a pair of closely related genes that grouped together with the AtSTY1/SHI/SRS8 clade (Niben101Scf00158g06008.1 and Niben101Scf08273g05002.1, **Figure 1**). For simplicity, we renamed these as *NbSRS-L1*, *NbSRS-L2*, and *NbSRS-L3* (**Figure 1**). In addition, we also chose for functional characterization the *EcSRS-L* gene that we were able to amplify from *E. californica* flowers. An alignment of all the sequences of the predicted proteins used in this study, also including the Arabidopsis AtSTY1, AtSRS3, AtSRS5, and EscaSTY-l factors is shown in **Supplementary Figure S1**.

### *SHI/STY/SRS* Gene Expression Patterns Are Similar in Arabidopsis, *E. californica*, and *N. benthamiana*

The expression pattern of the *SRS-L* genes identified in *E. californica* and *N. benthamiana* was characterized by RNA *in situ* hybridization on young flower buds.

**FIGURE 2:**
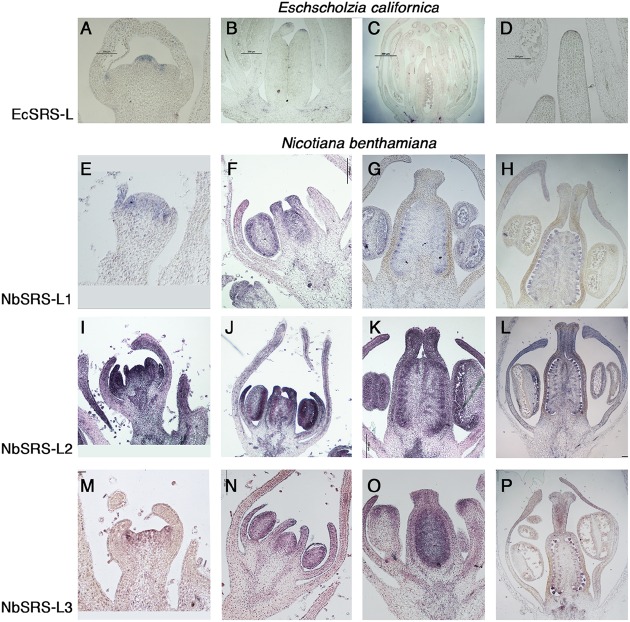
***In situ* expression analyses of *SRS-L* genes in *E. californica*, and *N. benthamiana flowers*.** Control hybridizations with sense probes are shown in **Supplementary Figure S2**. **(A–D)** Longitudinal sections of *E. californica* flowers probed with *EcSRS-L*. **(A)** At stage 3, *EcSRS-L* mRNA is detected in a few cells at the tip of the incipient pistil primordium and of sepal and petal primordia. **(B)** At stage 6, expression in the gynoecium is restricted to the most apical cells of the incipient style. **(C)** Later in development, expression of *EcSRS-L* is detected in ovule primordia, the anthers and in the most apical cells of the developing style. **(D)** Close up of the apical style shown in **(C)**. **(E–P)** Longitudinal sections of *N. benthamiana* flowers probed with *NbSRS-L* genes **(E–H)**, *NbSRS-L2* **(I–L)** and *NbSRS-L3* **(M–P)**. The three genes show remarkably similar expression patterns, although *NbSRS-L2* appears to be expressed at higher levels. **(E–H)** *NbSRS-L1* mRNA is detected in the floral primordium at very early stages **(E)**, and later it accumulates in the anthers, the developing placenta and the distal end of petals and carpels **(F)**. **(G,H)** After all floral organs differentiate, *NbSRS-L1* is mainly detected in ovule primordia, the style, the anthers and the distal end of growing petals. **(I,J)** *NbSRS-L2* is strongly expressed throughout flower development, mainly accumulating in distal positions in floral organs, placenta, ovules, anthers and the style. **(M,N)** The expression pattern of *NbSRS-L3* is very similar to that of *NbSRS-L1*, although the expression in the style appears stronger at later stages **(O,P)**.

*EcSRS-L* transcripts were weakly detected in young flowers from early stages of development. In very young *E. californica* buds, *EcSRS-L* was detected in the emerging stamen and carpel primordia (**Figure 2A**). At later stages, *EcSRS-L* mRNA accumulated weakly in anthers and in the placenta and ovules (**Figures 2B,C**). *EcSRS-L* expression could also be detected in the most distal cells of the developing gynoecium, in the presumptive domain that would further develop into the style and the stigma (**Figures 2B–D**). This expression pattern generally resembled those described for Arabidopsis *SHI/STY/SRS* genes, which showed distal accumulation in the developing gynoecium and, at least in the case of *AtSTY1*, expression in ovules (Kuusk et al., 2002).

In *N. benthamiana*, the three *SHI/STY/SRS* genes included in this study showed similar expression patterns, although not identical. *NbSRS-L1* was detected in very young floral buds, before floral organs in inner whorls were initiated (**Figure 2E**). At later stages, *NbSRS-L1* expression concentrated in the distal end of growing petals and of the gynoecium, as well as in the placentae and the anthers (**Figure 2F**). After style fusion, *NbSRS-L1* was mainly detected in ovules and pollen grains, while only weakly present in the transmitting tissues at the central domain of the style and in the stigma (**Figures 2G,H**). *NbSRS-L2* showed a more expanded expression in floral organs. In young buds, *NbSRS-L2* expression was associated to all incipient floral organ primordia (**Figure 2I**). At later stages, expression was detected in growing petals, stamens and gynoecia, mainly at the distal end (**Figure 2J**). After style fusion, *NbSRS-L2* was expressed mainly in ovules and it could be also found in the central domain of the style, the ovary wall and the distal end of growing petals (**Figures 2K,L**). *NbSRS-L3* expression pattern was most similar to that of *NbSRS-L1* (**Figures 2M–O**), although at later stages expression in the inner style was stronger than for *NbSRS-L1* (**Figure 2P**).

In summary, *NbSRS-L1, NbSRS-L2*, and *NbSRS-L3* genes showed remarkably similar expression patterns during flower development, being detected in all floral organ primordia from early stages of development and then mainly associated to the distal end of petals and carpels, anthers, placentae, and ovule primordia, as well as the inner style in more advanced stages. Again, these expression patterns resembled those described for several Arabidopsis *SHI/STY/SRS* genes (Kuusk et al., 2002, 2006).

### Overexpression of *NbSHI/STY/SRS* Genes in *Arabidopsis thaliana* Mimics the Effect of the Ectopic Expression of the Endogenous Arabidopsis *STY* Genes

**FIGURE 3:**
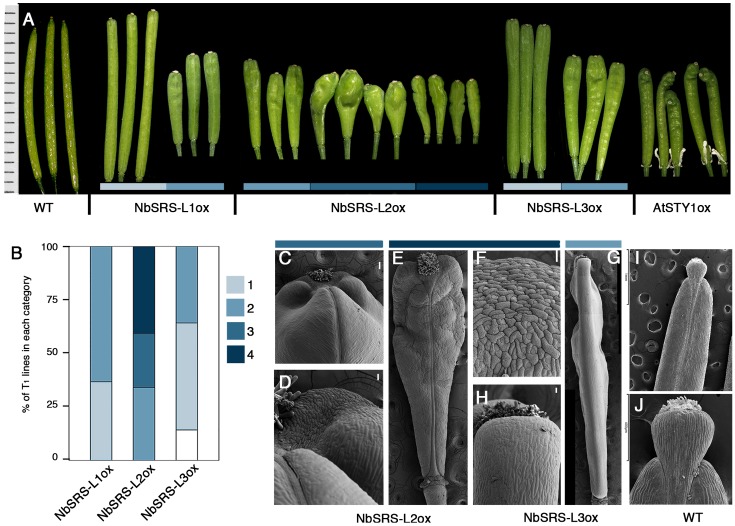
**Phenotypes of Arabidopsis transgenic lines overexpressing the *NbSRS-L* genes.** **(A)** Three to five fruits from a single plant representative of the different phenotypic categories and genotypes are shown. The AtSTYox fruits belong to a transgenic line obtained from E. Sundberg’s lab (Kuusk et al., 2002). **(B)** Proportions of the different phenotypic categories among the T1 transgenic lines obtained for each construct, where 1 (light blue) represents the weakest phenotype and 4 (dark blue) the strongest phenotype. White color corresponds to plants showing no conspicuous differences respect to wild type. **(C–J)** Scanning electron micrographs of fruits from selected genotypes and categories. **(C,D)** Fruits from 35S::NbSRS-L2 lines in category 3. **(C)** Apical part of the fruit. **(D)** Close up of the style/valve area. While cells retain their identity, there is no clear demarcation between the two zones. **(E)** Fruit from a 35S::NbSRS-L2 line in category 4. **(F)** Close up of the apical part of the fruit shown in **(E)**. All the cells have a crenelated surface with the typical morphology of style cells and no valve cells can be distinguished. **(G)** Lateral view of a 35S::NbSRS-L3 fruit in category 2. The valves grow laterally to the same level as the stigma and style and have a square morphology. **(H)** Close up of the apical valve cells of the fruit shown in panel **(G)**. All cells have valve identity and no ectopic formation of style cells can be observed. **(I)** Wild type fruit. **(J)** Close up of the apical domain, where the demarcation of stigma, style, and valves is clearly seen. Bars in **(C–G)**: 50 μm.

*Eschscholzia californica*, *N. benthamiana*, and Arabidopsis *SHI/STY/SRS* genes showed similar expression patterns, suggesting that they might have conserved functions. However, outside the RING and IGGH domains, low similarity could be found among *E. californica*, *N. benthamiana*, and Arabidopsis SHI/STY/SRS factors (**Supplementary Figure S1** and **Table S1**). Nevertheless, extensive functional redundancy of Arabidopsis genes has been shown in spite of sequence divergence (Kuusk et al., 2006), suggesting that the RING and IGGH domains might be sufficient for their shared function. To explore whether the *NbSRS-L* genes under study had similar functional properties among them and also to their Arabidopsis homologs, we transformed Arabidopsis wild type and *sty1 sty2* mutants with constructs for the overexpression of the three *NbSRS-L* genes and observed the corresponding phenotypes of at least 30 individual T1 plants for each construct and background. Overexpression of the three different *NbSRS-L* genes caused similar phenotypic alterations in carpel and fruit development when transformed into wild type Arabidopsis plants, resembling those observed for previously reported lines constitutively expressing *AtSTY1* or *AtSTY2* (Kuusk et al., 2002) (**Figure 3A**).

**FIGURE 4:**
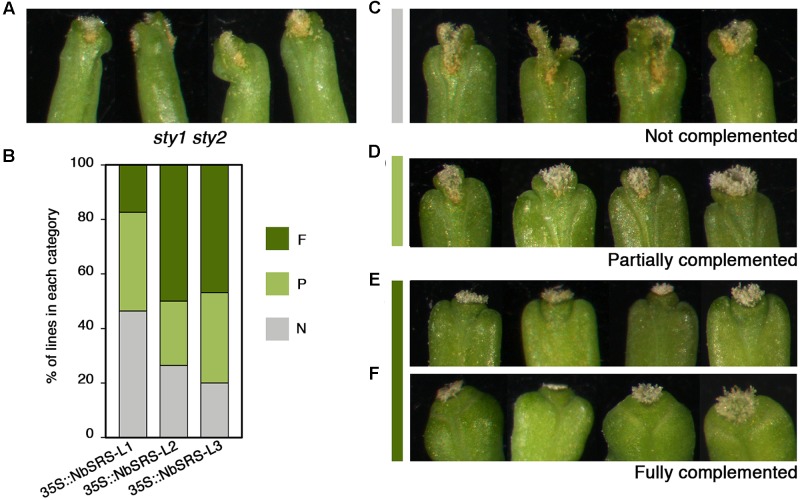
**Complementation phenotypes of Arabidopsis *sty1 sty2* mutants transformed with 35S::NbSRS-L constructs.** **(A)** Style and stigma phenotype of *sty1 sty2* mutants. **(B)** Proportions of the different phenotypic categories among the T1 transgenic lines obtained for each construct, where N (gray) represents non-complemented lines, P (light green) partially complemented lines and F (dark green) fully complemented lines. **(C–F)** Examples of the different complementation categories among the transgenic lines.

35S::NbSRS-L1 and 35S::NbSRS-L3 caused milder defects, mainly affecting the shape and length of the style, which was reduced and did not elongate as in wild type fruits, and the overall shape of the fruit, which appeared wider and blunt at the distal end in the lines with weaker phenotypic defects (**Figures 3A,B**, category 1), or shorter than wild type, wider specially at the apical end, and with an irregular ovary surface (**Figures 3A,B,G,H**, category 2) in the lines with stronger defects. *NbSRS-L2* overexpression caused related but more dramatic phenotypic alterations in fruit development. About one third of the lines showed fruit phenotypes resembling those of the most affected *NbSRS-L1* or *NbSRS-L3* overexpressors (**Figures 3A,B**), but most of the transgenic lines showed further enhanced defects that were subdivided in two additional categories (**Figures 3A,B**, categories 3 and **4**). Fruits in category 3 had short and inflated ovaries (**Figure 3A**). The cell in the valves had similar shapes to wild type but were not oriented in parallel to the apical-basal axis of fruit growth, but formed an angle with the replum (**Figures 3C,I**). In addition, the valve margins at the apical end were not clearly defined, the style was short and expanded laterally, and the demarcation between the style and the valves was not discernible (**Figures 3C,D,J**). Finally, about 40% of the 35S::NbSRS-L2 lines had short fruits of tapered shape, with short ectopic style cells developing extensively at the lateral domains of the apical half of the valves (**Figures 3E,F**), and cells closer to the replum that retained valve identity but elongated forming a wider angle with the apical-basal axis (**Figure 3E**). When the same constructs were introduced into Arabidopsis *sty1 sty2* mutants, all of them were able to partially or fully rescue the phenotypic defects of *sty1 sty2* stigma and style (**Figures 4A–F**), although at different degrees.

Altogether, these results indicated that the three *N. benthamiana* SHI/STY/SRS factors under study had similar molecular properties among them and also, at least, to the Arabidopsis *STY1* or *STY2* genes in spite of high sequence divergence outside the RING and IGGH domains.

A similar approach was undertaken with the *EsSRS-L* gene identified in this study and 30 independent 35S::EcSRS-L T1 lines were generated in wild type and *sty1 sty2* backgrounds. However, *EcSRS-L* overexpression was not able to produce neither any phenotypic effect in the wild type background, nor complementation of the *sty1 sty2* mutant phenotype (**Supplementary Figure S3**).

### Silencing of *NbSHI/STY/SRS* Genes in *Nicotiana benthamiana* Using VIGS Alters Floral Organ Growth and Style and Stigma Development

The results of overexpressing the three *NbSRS-L* genes in Arabidopsis wild type or *sty1 sty2* plants suggested that the Nicotiana NbSRS-L factors were functionally equivalent to AtSTY1 or AtSTY2 to a large extent, but did not clarify whether these genes had similar roles in Nicotiana development to those described for the *SHI/STY/SRS* genes in Arabidopsis. To investigate the function of the three *NbSRS-L* genes in this study, we used Virus Induced Gene Silencing (VIGS) to reduce their transcript levels (Ratcliff et al., 2001; Wege et al., 2007; Fourquin and Ferrandiz, 2012). We generated three different TRV constructs designed to downregulate *NbSRS-L1*, *NbSRS-L2*, or *NbSRS-L3*. Twelve plants were inoculated with each construct independently and 5–20 flowers from each plant were chosen for further characterization (**Table 1**).

**Table 1:** Summary of the VIGS experiments on *Nicotiana benthamiana* and *Eschscholzia californica* plants.

VIGS construct	N° plants inoculated (dead)	N° plants with phenotype	N° flowers/plant observed	N° flowers/plant with phenotype	N° total flowers with phenotype	% Flowers with phenotype
*N. benthamiana*						
TRV2-NbSRS-L1	12 (5)	7	10–20	4–14	82/128	64%
TRV2-NbSRS-L2	12 (2)	8	7–20	1–6	22/143	15%
TRV2-NbSRS-L3	12 (2)	9	5–20	2–8	30/137	22%
TRV-NbSRS-L1/2	12 (3)	9	15–20	8–18	120/172	70%
TRV-NbSRS-L1/2/3	12 (3)	10	3–20	2–14	63/99	64%
Empty vector	6 (1)	0	20	0	0/100	0%
*E. californica*						
TRV2-EcSRS-L	120 (11)	0	1–5	0	0	0%
TRV2-EcPDS	70 (22)	48	n.d.	n.d.	48/70^∗^	68%^∗^
Empty vector	60 (16)	0	2	0	0/88	0%


*^∗^Bleaching caused by PDS inactivation.*

**FIGURE 5:**
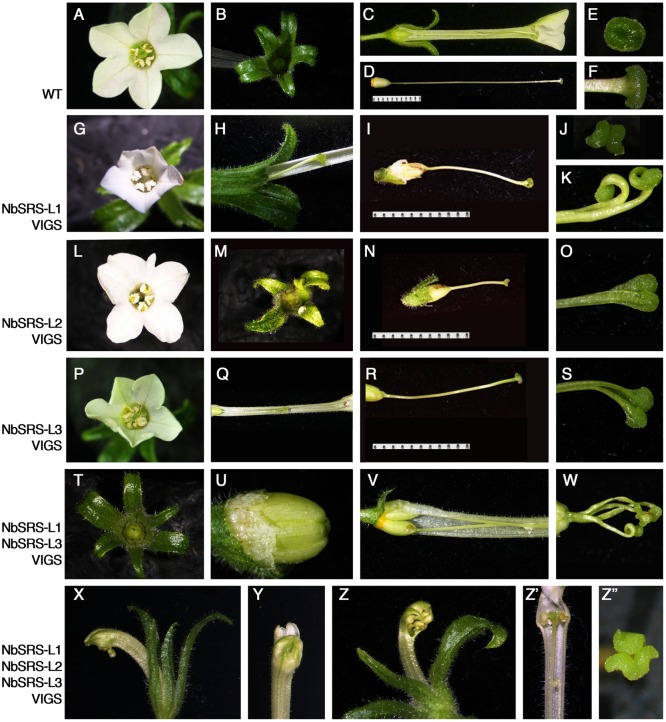
**Phenotypes of *N. benthamiana* plants inoculated with different pTRV2-NbSRS-L constructs.** **(A–F)** Wild type *N. benthamiana* flower at anthesis. **(A)** Top view. Five expanded white petals and the central stigma surrounded by five stamens are clearly seen. **(B)** Top view of the dissected calix, formed by five green narrow sepals. **(C)** Lateral view of the flower, where some petals al sepals have been removed to show the ovary and the long style, that ends in a stigma leveled with the anthers. **(D)** Lateral view of the gynoecium. **(E,F)** Top and lateral view of the stigma. **(G)** NbSRS-L1-VIGS flower. The petals are reduced in size and the stigma is not visible at the center. **(H)** Lateral view of the basal part of the flowers, where some petals have been removed, to expose the short style. **(I)** NbSRS-L1-VIGS gynoecium. **(J,K)** Two examples of the defects found in the stigmas of NbSRS-L1-VIGS pistils. **(L)** NbSRS-L2-VIGS flower. The corolla is not symmetrical because one petal is severely underdeveloped. The stigma is not visible. **(M)** Top view of the dissected calix of a NBSRS-L2-VIGS flower. One of the sepals is reduced in size and deformed. **(N)** NbSRS-L2-VIGS gynoecium. **(O)** Close-up of the stigma shown in **(N)**, where the abnormal shape is visible. **(P)** Top view of a NbSRS-L3-VIGS flower. Note the unexpanded greenish corolla. **(Q)** Lateral view of a NbSRS-L3-VIGS flower where some petals have been removed to expose the short style. **(R)** Lateral view of a NbSRS-L3-VIGS gynoecium. **(S)** Example of defects observed in NbSRS-L3-VIGS styles. **(T–W)** Indeterminate phenotypes of flowers treated with TRV2-NbSRS-L1 and TRV2-NbSRS-L3 constructs simultaneously. **(T)** 6-sepal calix. **(U)** 4-carpel ovary. **(V)** Bi-pistillate flower. **(W)** Four style-like protrusions in a 4-carpel pistil. **(X–Z”)** Flowers inoculated simultaneously with the three TRV2-NbSRS-L constructs. **(X–Z)** Examples of mature flowers with severely unexpanded corollas. **(Z’)** Lateral view of a flower where a defective anther is visible. **(Z”)** Split and supernumerary stigma.

*Nicotiana benthamiana* has pentamerous flowers, where the perianth is composed of a calyx of five sepals, and five fused petals forming a tubular corolla. The five stamens are epipetalous and the gynoecium is bicarpellate and formed by a short bilocular ovary with central placentation and an elongated style, measuring more than 3 cm at anthesis, capped by a round flat stigma (**Figures 5A–F**). Plants inoculated with any of the three TRV2-NbSRS-L vectors showed similar phenotypic alterations, although TRV2-NbSRS-L1 had a greater efficiency (64% of the flowers showed abnormal development) when compared with TRV2-NbSRS-L2 (15%) or TRV2-NbSRS-L3 (22%) (**Table 1**). These defects affected upper corolla development, and most frequently consisted of alterations in the shape, the color or the symmetry of the petal lobes (**Figures 5G,L,P,M**). Style and stigma development were also strongly affected. Style length was reduced, and very frequently the style was unfused or even split at the apical end, where the stigma adopted irregular shapes (**Figures 5H–K,N,O,Q–S**). Aiming to enhance the phenotypic defects caused by *NbSRS-L* inactivation, 12 plants were also inoculated simultaneously with TRV2-NbSRS-L1/TRV2-NbSRS-L3 vectors or with TRV2-NbSRS-L1/TRV2-NbSRS-L2/TRV2-NbSRS-L3 vectors. These combined treatments produced phenotypic effects in 70 and 64% of the observed flowers, respectively, therefore at levels comparable to those resulting of TRV2-NbSRS-L1 inoculation alone. However, in addition to the phenotypes already described for the individual constructs, these combinations induced novel alterations mainly related to floral organ number. In plants treated with TRV2-NbSRS-L1/TRV2-NbSRS-L3, the calix was frequently formed by six sepals (**Figure 5T**). Gynoecia formed by four fused carpels were also observed, sometimes even capped by multiple styles derived from each carpel (**Figures 5U,W**), while in other flowers two bicarpellated fused pistils were formed (**Figure 5V**). Simultaneous inoculation with the three vectors did not further enhance the defects observed in TRV2-NbSRS-L1/TRV2-NbSRS-L3 treated plants (**Figures 5X–Z”**), except for occasional development of abnormal anthers (**Figure 5Z**’) and stronger defects in corolla development (**Figures 5X–Z**).

The similar effect of all the VIGS treatments tested suggested that the three *NbSRS-L* genes under study had related functions, an idea already supported by the results of their heterologous constitutive expression in Arabidopsis. However, since it was likely that these related functions were redundant, it was surprising that the inoculation with combinations of different constructs did not dramatically enhance the associated phenotypic defects, suggesting that the different TRV2-NbSRS-L vectors could be targeting more than one gene in the family and therefore inducing transitory inactivation of several *NbSRS-L* genes at once. The efficiency and specificity of each VIGS treatment was assessed by measuring the level of expression of *NbSRS-L1*, *NbSRS-L2*, and *NbSRS-L3* on flowers from two treated plants for each treatment that showed inactivation-related phenotypes. The results from these experiments showed that the VIGS treatments were effective in reducing the expression levels of the *NbSRS-L* genes, but that they did not achieve a high degree of specificity and therefore the observed phenotypes were likely caused by simultaneous inactivation of several *NbSRS-L* genes (**Supplementary Figure S4**).

The effect of transient inactivation of *EcSRS-L* in *E. californica* was also tested by inoculation of plants with a TRV2-EcSRS-L construct. One hundred and twenty plants were inoculated but none of them showed evident phenotypic defects (**Table 1**), in spite of the effective inactivation caused by the VIGS treatment as quantified by qRT-PCR (**Supplementary Figure S5**), indicating that either the *EcSRS-L* gene was fully redundant with the *EscaSTY* gene described in (Pfannebecker et al., 2017a), that the residual expression of *EcSRS-L* in VIGS treated plants was sufficient to provide its function, or that the phenotype associated with *EcSRS-L* inactivation was not affecting morphogenesis in a conspicuous way.

### Overexpression of *NbSHI/STY/SRS* Genes in *Nicotiana benthamiana* Cause Ectopic Style and Stigma Development

**FIGURE 6:**
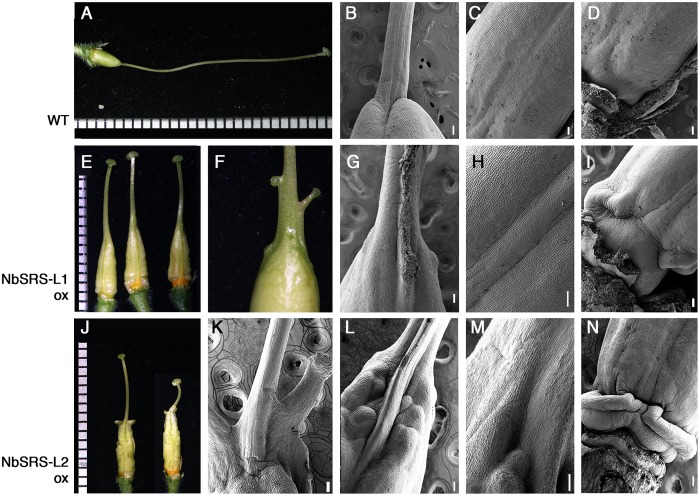
**Phenotypes of pistils of transgenic *N. benthamiana* lines overexpressing *NbSRS-L1* or *NbSRS-L2* genes.** **(A–D)** Wild type pistils. **(A)** Lateral view. **(B–D)** Scanning Electron Micrographs of different parts of the wild type pistil. **(B)** Style-ovary junction. **(C)** Medial view of the ovary. The valve margins are adjacent and appear as a subtle crease at the center. **(D)** View of the gynophore at the basal end of the ovary. **(E–I)** 35S::NbSRS-L1 pistils. **(E)** Three pistils from different transgenic plants showing similar phenotypes. **(F)** Close-up of the ovary-style junction. Small protuberances capped with stigmatic tissue can be observed. **(G–I)** Scanning Electron Micrographs of 35S::NbSRS-L1 pistils. **(G)** Close-up of the ovary-style junction. Note the poor delimitation of both domains and the formation of ectopic stigmatic tissue. **(H)** Medial view of the ovary. The valve margins, which appear as constricted longitudinal ridges, leave a bulging area of several cell files between them resembling the Arabidopsis replum. **(I)** View of the elongated gynophore of a 35S::NbSRS-L1 pistil. **(J–N)** 35S::NbSRS-L2 pistils. **(J)** Two pistils from different transgenic plants showing similar phenotypes. **(K–N)** Scanning Electron Micrographs of 35S::NbSRS-L1 pistils. **(K)** Close-up of the ovary-style junction. Note the expansions of style tissue capped with stigmatic cells. **(L)** Close-up of the ovary-style junction of a different pistil. Note the bulging protrusion in medial position and the highly irregular ovary surface. The demarcation of style and ovary is not defined. **(M)** Close-up of another style-ovary junction where the mixed identity of style and ovary cells is observed. **(N)** View of the gynophore, where abnormal development of tissue is observed. Bars in micrographs: 100 μm.

To study the effect of ectopic expression of the *NbSRS-L* genes under study in *N. benthamiana* we generated transgenic plants in which we introduced the 35S::NbSRS-L1 and 35S::NbSRS-L2 constructs previously used for heterologous expression in Arabidopsis. Twenty-three independent transgenic lines were obtained for 35S::NbSRS-L1 and 9 for 35S::NbSRS-L2, and around half of them showed conspicuous phenotypic changes in gynoecium morphology (**Figures 6A,E,J**). Both 35S::NbSRS-L1 and 35S::NbSRS-L2 plants showed similar phenotypes, although, as already observed in Arabidopsis, the constitutive expression of N*bSRS-L2* had more dramatic effects.

**FIGURE 7:**
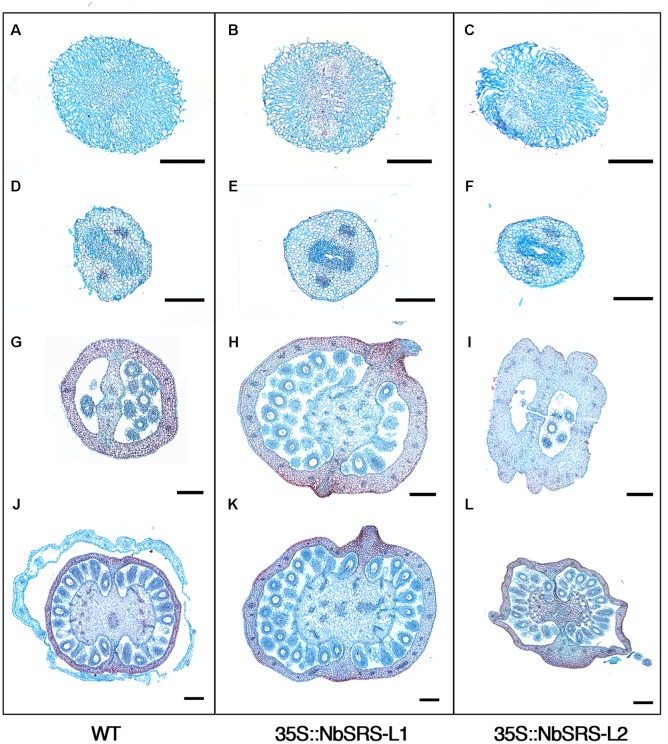
**Phenotypic characterization of *N. benthamiana* pistils from 35S::NbSRS-L1 and 35S::NbSRS-L2 lines.** Histological sections of anthesis pistils. **(A,D,G,J)** Wild type. **(B,E,H,K)** 35S::NbSRS-L1. **(C,F,I,L)** 35S::NbSRS-L2. **(A–C)** Transversal sections of stigmas. Note the similar morphology in all cases. **(D–F)** Transversal sections of the style. Again, style morphology appears similar in all three genotypes. **(G–I)** Transversal sections at the distal portion of the ovary. Note the medial protrusions capped with stigmatic tissue in 35S::NbSRS-L1 **(H)** and the engrossed and irregular ovary walls in 35S::NbSRS-L2 **(I)**. **(J–L)** Transversal sections of the basal domain of the ovary. The morphology of 35S::NbSRS-L pistils is more similar to wild type, but still medial protrusions and additional cell layers in the ovary wall can be observed in 35S::NbSRS-L1 **(K)** and the ovary walls are irregular in shape in 35S::NbSRS-L2 **(L)**. Bars: 200 μm.

The pistils of plants overexpressing *NbSRS-L1* had stigma and style of normal morphology, only shorter (**Figure 6E**). The demarcation between style and ovary was not defined as in wild type (**Figure 6B**), but showed a gradual transition between the elongated zone with columnar cells typical of the style and the nearly isodiametric small cells of the ovary (**Figures 6E–G**). The valve margins in the ovary, which in the wild type are poorly defined and adjacent (**Figure 6C**), in the 35S::NbSRS-L1 pistils appeared more pronounced and separated by several cell files, that in the upper part of the ovary bulged forming ridges capped by stigmatic cells (**Figures 6E–H**). Occasionally, small cylindrical protrusions, similar to styles and terminated by a rounded stigma, grew out of these ridges (**Figure 6F**). Finally, the gynophore was elongated and showed a clear demarcation from the ovary (**Figures 6D,I**). 35S::NbSRS-L2 pistils showed similar phenotypes, only enhanced. The junction between style and ovary showed stronger alterations and more frequently gave rise to what appeared to be ectopic styles (**Figures 6J–L**). The surface of the ovary was irregular and grew unevenly, with intermixed domains of different style/ovary cell identity (**Figures 6J,L,M**). The gynophore was also more pronounced and separated from the ovary by a bulging ridge of tissue (**Figure 6N**). Anatomical sections confirmed that the style and stigma of the transgenic lines were similar to wild type (**Figures 7A–F**). The ovaries of the lines overexpressing *NbSRS-L1* had thickened walls with between 2 and 4 more cell layers in the mesocarp than the wild type, and the ridges bulging at the valve margins clearly showed mixed identity of ovary and style/stigma tissues (**Figures 7G,H,J,K**). In the 35S::NbSRS-L2 lines, the upper part of the ovary showed thickened and irregular walls that did not resemble clearly the ovary wall morphology of the wild type (**Figure 7I**); the basal part of the ovary was less affected, but still showed irregularities in shape (**Figure 7L**).

**FIGURE 8:**
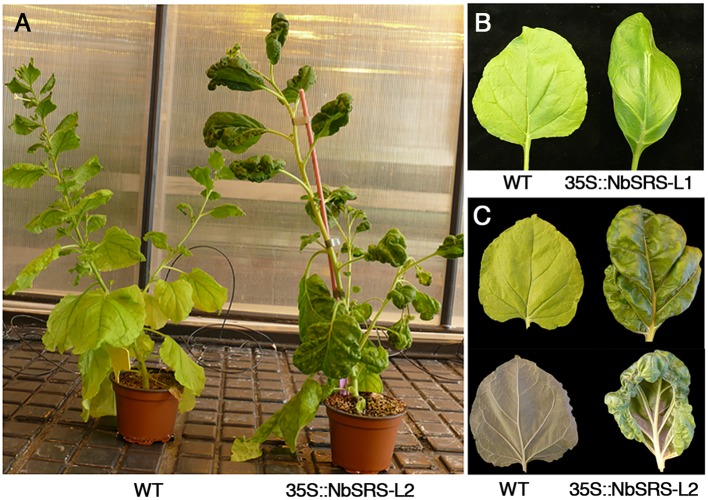
**Overexpression of *NbSRS-L* genes in *N. benthamiana* alters leaf development by constricting margin expansion.** **(A)** Wild type (left) and 35S::NbSRS-L2 (right) plants of similar age. The 35S::NbSRS-L2 plant is darker, has slightly increased internode length and aberrant leaf shape. **(B)** Adaxial view of a fully expanded wild type (left) and a 35S::NbSRS-L1 (right) leaf. The leaf margin in the transgenic plant does not expand to the same extension as the blade, and the leaf is no longer flat. **(C)** Adaxial (top) and abaxial (bottom) views of a wild type (left) and a 35S::NbSRS-L2 (right) leaf. Note the dramatically constricted leaf margin of the transgenic leaf, which shows a much darker color and a highly irregular surface.

In addition to changes in gynoecium morphology, overexpression of *NbSRS-L* genes caused strong alterations in leaf development, especially in 35S::NbSRS-L2 lines. The leaves of the transgenic plants were darker than wild type and growth was constricted in the margins, causing the blade to adopt a hood-like appearance (**Figure 8**).

## Discussion

In this work, we have studied the functional conservation of members of the *SHI/STY/SRS* gene family in the asterid core eudicot *N. benthamiana* and the basal eudicot *E. californica*. We have determined the expression patterns of the genes under study during flower development, characterized the floral phenotypes caused by their downregulation, and the effects of their overexpression both in the heterologous system *A. thaliana* and in *N. benthamiana*. Our work supports the conserved function of S*TY/SHI/SRS* genes in apical gynoecium development and their position at the top of the GRN directing style and stigma development in core eudicots.

### *SHI/STY/SRS* Function in Apical Gynoecium Development Is Conserved in Core Eudicots

The *SHI/STY/SRS* genes of Arabidopsis act redundantly to specify style and stigma development, as revealed by the phenotype of multiple combinations of mutations in members of the family (Kuusk et al., 2006). The expression of different *SHI/STY/SRS* genes in Arabidopsis has been analyzed by mRNA *in situ* hybridization or the use of promoter::GUS reporters. These experiments show largely overlapping expression patterns, generally associated with domains of auxin accumulation in all lateral organs and in the apical tissues of the developing gynoecium, consistent with the high level of redundancy found in the family (Fridborg et al., 2001; Kuusk et al., 2002, 2006; Eklund et al., 2011). In this work, we have characterized the expression of a *SHI/STY/SRS* gene from the basal eudicot *E. californica* and of three members of the family from the core eudicot *N. benthamiana*, observing a significant conservation in the pattern of mRNA accumulation in the flowers of these two species and of Arabidopsis. In all cases, we have detected expression in the distal domain of growing floral organs in young floral buds, the ovule primordia and the apical domain of the pistil, indicating that the regulatory regions of these genes contain conserved elements, despite the evolutionary distance of the corresponding species and the differences among paralogs within the same genome. Interestingly, it has been shown that a regulatory element present in the promoters of most Arabidopsis S*HI/STY/SRS* genes, a GCC-box bound by transcription factors of the AP2/ERF family, is required for the expression of *SHI/STY/SRS* genes in the distal domain of lateral organs including the apical tissues of the developing pistils (Eklund et al., 2011). A recent work which includes a comprehensive phylogeny of the *SHI/STY/SRS* family also reports the search for GCC-boxes in the promoters of selected genes from this study, finding that it is present in almost all of the angiosperm sequences but not in more basal taxa such as those including mosses, lycophytes or conifers (Pfannebecker et al., 2017a). Not surprisingly, we could also detect conserved GCC-boxes within the 1 kb upstream promoter sequences of the three Nicotiana genes, for which genomic sequences were available, supporting their importance in mediating apical gynoecium expression.

Heterologous constitutive expression of *N. benthamiana SHI/STY/SRS* genes in Arabidopsis caused phenotypic effects similar to those of overexpressing the endogenous genes (Kuusk et al., 2002; Kim et al., 2010), including ectopic style tissue formation, loss of definition between the style and the ovary, and an overgrowth of the valves at the apical end of the pistil, a morphology typically associated to auxin accumulation and that mimics exogenous treatment with auxin (Ståldal et al., 2008). Despite the sequence divergence between the sequences of Arabidopsis and Nicotiana proteins, which is high outside the conserved RING and IGGH domains, and the occurrence of independent duplication events in each species, the overexpression phenotypes associated to the three *NbSRS-L* genes were similar, as well as the ability of the different NbSRS-L factors to complement the *sty1 sty2* mutant phenotype. These experiments support that many of the SHI/STY/SRS proteins are basically equivalent in molecular function, even if outside the conserved RING and IGGH domains they show low sequence similarity, suggesting that most of their interactions with DNA targets and other proteins would be mediated by these conserved domains. In this context, it was even more surprising that the *EcSRS-L* gene did not complement the Arabidopsis *sty1 sty2* mutant phenotype, neither caused phenotypic defects when overexpressed in Arabidopsis wild type plants. A close inspection of the RING and IGGH domains showed high sequence similarity with that of other members of the family, although the last Cys residue in the zinc finger domain is preceded by a Pro residue in the *E. californica* proteins while in most members of the family this position is most frequently a charged or polar residue like Asp, Glu, His, or Gln. Given the structural properties of Pro residues, which have an exceptional conformational rigidity that usually impacts protein secondary structure and protein–protein interactions (Morris et al., 1992), it is possible that this difference may affect function. This Pro residue is not exclusive of the *E. californica* homologs, but it was also found in 5 of the 91 predicted proteins included in the phylogenetic study by Pfannebecker et al. (2017a). However, since none of these homologs has been functionally characterized, it would be necessary to perform equivalent analyses with some of them to test this hypothesis.

Silencing of the *NbSRS-L* homologs by VIGS also supported their conserved role in style and stigma development at least in *N. benthamiana*. The pistils of VIGS-NbSRS-L plants displayed a range of phenotypic defects that affected strongly these tissues, mostly shortening of the style and split and abnormal stigma formation. In addition, other phenotypic defects associated previously with loss of SHI/STY/SRS function in Arabidopsis, like general defects in floral organ growth and anther development, were also observed (Kuusk et al., 2006). We found the VIGS treatments not to be specific among the *NbSRS-L* genes under study, so it is not possible to estimate the level of genetic redundancy or the specific functions of each individual gene. However, since the three of them show similar patterns of expression and cause similar effects when overexpressed in Arabidopsis or in Nicotiana, it seems most likely that they work redundantly as described for SHI/STY/SRS family members in Arabidopsis. Unfortunately, the EcSRS-L-VIGS treatments were not sufficient to induce phenotypic changes in *E. californica*, so we cannot infer any significant conclusion. The lack of abnormal phenotypes in VIGS-treated plants and in the Arabidopsis plants expressing *EcSRS-L* may suggest that the EcSRS-L protein is not functional and that other members of this or other families provide the style-stigma specification function. Only one additional *SHI/STY/SRS* gene has been described in *E. californica*, which presents high level of homology with the *EcSRS-L* gene in this study, including the distinctive Pro residue in the RING domain and thus unlikely to be functionally divergent (Pfannebecker et al., 2017a). However, the *E. californica* genome has not been sequenced yet, and it is possible that it encodes other members of the family that may have a role in style and stigma development. It would be interesting to extend this type of studies to other basal dicots and even more basal taxa within angiosperms to conclusively support the conservation of the role of SHI/STY/SRS factors in style and stigma differentiation across angiosperms. However, the reports of abnormal style and stigma development in the monocot *Hordeum vulgare short awn* mutants, affected in the *SHI/STY/SRS* gene *Lks2*, strongly support this idea (Yuo et al., 2012).

NbSRS-L VIGS-treated plants in Nicotiana occasionally showed increased number of floral organs or multicarpellate pistils. These phenotypes had not been previously reported for *shi/sty* mutants in Arabidopsis, and could reflect a specific function for *SHI/STY/SRS* genes in Nicotiana. However, it has been recently reported that the *vrs2* mutants in barley, affected in a *SHI/STY/SRS* gene, form supernumerary spikelet meristems in the inflorescence (Youssef et al., 2017), an indeterminate behavior somehow reminiscent of the supernumerary floral organs found in VIGS-NbSRS-L plants and that could reveal an additional conserved role of the members of the SHI/STY/SRS family, that might be related to their described conserved function in hormone homeostasis (Fridborg et al., 2001; Eklund et al., 2010b, 2011; Kim et al., 2010; Zawaski et al., 2011; Youssef et al., 2017).

### A Conserved GRN for Carpel Development

The phenotypes of Nicotiana 35S::NbSRS-L lines parallel those described for Arabidopsis 35S::STY1 plants. In addition to support functional conservation among *SHI/STY/SRS* genes at the top of the GRN involved in style and stigma specification, these results also suggest that the set of targets for these factors in both species is also very conserved. Thus, the downstream pathways leading to style and stigma development are probably similar, despite the dramatic differences of style and stigma morphologies in both species. Moreover, in addition to directing ectopic development of these tissues, the overall changes in pistil morphology, such as the less conspicuous demarcation between ovary and style, or the irregular proliferation of cells in the ovary walls causing crinkled ovary surface, suggest that the process of patterning the pistil into different territories and functional domains responds to similar cues in species with highly diverse pistil morphologies.

Our study also reveals that, as in Arabidopsis, *NGA* and *SHI/STY/SRS* genes also share their functions in pistil development in Nicotiana. *NbNGA* and *NbSRS-L* genes show very similar expression patterns in flower development and lead to highly related phenotypes when downregulated by VIGS (Fourquin and Ferrandiz, 2014). However, while *NbNGA* inactivation caused a complete lack of stigma and style tissues in Nicotiana pistils, *NbSRS-L* downregulation only produced milder defects in these same tissues, which could suggest that the contribution of NbSRS-L factors to this function is less important. However, VIGS treatments were not as efficient for *NbSRS-L* inactivation as for the reported VIGS-NbNGA studies, and the number of *SHI/STY/SRS* genes in the *N. benthamiana* genome is also higher than the number of *NbNGA* homologs, and therefore it is likely that the observed phenotypes of the NbSRS-L-VIGS plants do not reflect the effect of a full loss of SHI/STY/SRS function (Fourquin and Ferrandiz, 2014). Interestingly, the phenotypes of *N. benthamiana* 35S::NbSRS-L lines also resemble some aspects of the effect of overexpressing the *AtNGA* genes in Arabidopsis, such as an enlarged and bulging replum (the region between the valve margins) or the elongation of the gynophore, also supporting the conserved position of both classes of factors at the top of the GRN directing style and stigma development where they would converge in common targets and functions (Trigueros et al., 2009).

*NGA* genes belong to the RAV subfamily of B3-domain transcription factors present in all land plants, but they possess three characteristic conserved domains only found in angiosperm NGA proteins (Fourquin and Ferrandiz, 2014; Pfannebecker et al., 2017b). *SHI/STYSRS* homologs are found already in bryophytes, although the GCC-box conferring carpel expression has only been found in the promoters of angiosperm *SHI/STY/SRS* genes (Pfannebecker et al., 2017a). This suggests that the *NGA* and the *SHI/STY/SRS* genes may have acquired angiosperm-specific functions linked to the evolutionary origin of the style and the stigma, angiosperm-specific tissues themselves.

In addition to *NGA* and *SHI/STY/SRS* genes, other factors involved in style and stigma development have been shown to have conserved functions in several angiosperm species. Members of the PLE-subclade of MADS-box genes have been related to style and stigma development in different dicot species (Colombo et al., 2010; Fourquin and Ferrandiz, 2012; Heijmans et al., 2012). Likewise, orthologs of *CRABS CLAW (CRC)* gene, a member of the YABBY family of transcription factors required for correct style development and apical gynoecium closure in Arabidopsis, also have a conserved role in these functions in a wide range of angiosperm species (Yamaguchi et al., 2004; Fourquin et al., 2005, 2007, 2014; Lee et al., 2005; Ishikawa et al., 2009; Orashakova et al., 2009; Yamada et al., 2011). Taking all this evidence together, we could propose an emerging evolutionary conserved GRN directing carpel patterning which would include *NGA*, *SHI/STY/SRS, CRC*, and *PLE*-like genes as a core of factors linked to style and stigma development, although we still need to investigate in more depth the conservation of their regulatory relationships. In addition, it would be interesting to expand these studies to other genes with a described role in apical pistil development in Arabidopsis, such as *HECATE*, *NO TRANSMITTING TRACT* or *HALF FILLED* (Crawford et al., 2007; Gremski et al., 2007; Crawford and Yanofsky, 2011), which have not been functionally characterized in other taxa, and to connect this apical gynoecium network with those directing valve margin, dehiscence or fruit patterning, which also have been addressed lately (Ferrandiz and Fourquin, 2014; Pabon-Mora et al., 2014).

## Materials and Methods

### Plant Material and Growth Conditions

*Eschscholzia californica* Cham. and *Nicotiana benthamiana* L. plants were grown in the greenhouse, at 22°C: 18°C (day : night), with a 16 : 8 h, light : dark photoperiod, in soil irrigated with Hoagland no. 1 solution supplemented with oligoelements (Hewitt, 1966). *E. californica* germplasm used in this study (accession no. PI 599252) was obtained from the National Genetic Resources Program (United States).

For Arabidopsis transformation, *EcSRS-L* and *NbSRS-L* coding sequences were amplified with primers EcSTYF/EcSTYR (EcSRS-L), NbSTY1For/NbSTY1Rev (NbSRS-L1), NbSTY2For/NbSTY2Rev (NbSRS-L2), NbSTY3For/NbSTY3Rev (NbSRS-L3), cloned into PCR8/GW/TOPO (Invitrogen) and then transferred by Gateway reactions into the pMCD32 destination vector (Curtis and Grossniklaus, 2003). Each vector was introduced into *Agrobacterium tumefaciens* PMP90 for Arabidopsis transformation using the floral dip protocol (Clough and Bent, 1998) into the wild type Columbia background or the *sty1-1 sty2-1* double mutants (Kuusk et al., 2002). T1 plants were selected based on kanamycin selection. *N. benthamiana* transformation was done using the same vectors and following previously described standard procedures (Clemente, 2006). Three to five T1 individuals from each transformation were tested to confirm that the transgene was being expressed by qRT-PCR. See **Supplementary Table S2** for primer sequences.

### Cloning and Sequence Analysis

The partial coding sequence of *EcSRS-L* gene was isolated by RT-PCR on cDNA of flowers of *E. californica* using the degenerate primers EcSTYdF2/EcSTYdR2 designed from the conserved motifs of SRS homologs from other species. The 3′ end of EcSRS-L was then isolated by reverse transcription polymerase chain reaction (RT-PCR) using the primers PoppyRT and RT (sequence added to the oligodT primer used for retrotranscription). To amplify the *NbSRS-L* genes, we designed primers from the annotated genomic sequences retrieved from solgenomics.net (same pairs as described for cloning the CDS in the previous subheading).

The deduced amino acid sequences alignments were analyzed using the MUSCLE tool built in the Macvector 15.1 software (MacVector Inc., Cary, NC, United States). The phylogenetic tree was inferred using the Neighbor-Joining method (Saitou and Nei, 1987). The optimal tree with the sum of branch length = 4.39003061 is shown. The percentage of replicate trees in which the associated taxa clustered together in the bootstrap test (2000 replicates) are shown next to the branches. The tree is drawn to scale, with branch lengths in the same units as those of the evolutionary distances used to infer the phylogenetic tree. The evolutionary distances were computed using the p-distance method and are in the units of the number of amino acid differences per site. The analysis involved 24 amino acid sequences. All ambiguous positions were removed for each sequence pair. There was a total of 723 positions in the final dataset. Evolutionary analyses were conducted in MEGA7 (Kumar et al., 2016).

### *In Situ* Hybridization

RNA *in situ* hybridization with digoxigenin-labeled probes was performed on 8 μm paraffin sections of *E. californica* and *N. benthamiana* buds, as described by Ferrándiz et al. (2000). The RNA antisense and sense probes were generated from a 294 bp fragment of the EcSRS-L cDNA (positions 1-294), from a 438 bp fragment of the NbSRS-L1 cDNA (positions 607-1045), from a 407 bp fragment of the NbSRS-L2 cDNA (positions 1-407) and from a 536 bp fragment of the NbSRS-L3 cDNA (positions 1-536). Each fragment was cloned into the pGemT-Easy vector (Promega), and sense and antisense probes were synthesized using the corresponding SP6 or T7 polymerases.

### Virus-Induced Gene Silencing (VIGS)

The same regions of EcSRS-L, NbSRS-L1, and NbSRS-L3 coding sequences used for *in situ* hybridization were used for the VIGS experiments, while for NbSRS-L2 a fragment of 537 bp (positions 1-537) was used. A Xba1 restriction site was added to the 5′ end of the PCR fragment and a BamH1 restriction site was added to the 3′ end. The amplicon was digested by Xba 1 and Bam H1 and cloned into a similarly digested pTRV2 vector. The resulting plasmids were confirmed by digestion and sequencing, before being introduced into the *Agrobacterium tumefaciens* strain GV3101. The agroinoculation of *E. californica* seedlings was performed as described (Pabon-Mora et al., 2012). The agroinoculation of *N. benthamiana* leaves was performed as previously described (Dinesh-Kumar et al., 2003).

### Quantitative RT-PCR

Total RNA was extracted from flowers in anthesis with the RNeasy Plant Mini kit (Qiagen). Four micrograms of total RNA were used for cDNA synthesis performed with the First-Strand cDNA Synthesis kit (Invitrogen) and the qPCR master mix was prepared using the iQTM SYBR Green Supermix (Bio-Rad). The primers used to amplify EcSRS-L1 (qEcSTYFor and qEcSTYRev), NbSRS-L1 (qNbSTY1For and qNbSTY1Rev), NbSRS-L2 (qNbSTY2For and qNbSTY2Rev), and NbSRS-L3 (qNbSTY3For and qNbSTY3Rev) did not show any cross-amplification. Results were normalized to the expression of the *ACTIN* gene of *E. californica*, amplified by EcACTFor and EcCTRev, and to the Elongation Factor 1 (*EF1*) gene of *N. benthamiana* (accession no. AY206004), amplified by qNbEF1For and qNbEF1Rev (Fourquin and Ferrandiz, 2014). The efficiencies in the amplification of the genes of interest and the corresponding reference gene were similar. Three technical and two biological replicates were performed for each sample. The PCR reactions were run and analyzed using the ABI PRISM 7700 Sequence detection system (Applied Biosystems Inc., Life Technologies Corp., Carlsbad, CA, United States). See **Supplementary Table S2** for primer sequences.

### Scanning Electron Microscopy (SEM) and Histology

Plants treated with VIGS were analyzed by cryoSEM on fresh tissue under a JEOL JSM 5410 microscope equipped with a CRIOSEM instrument CT 15000-C (Oxford Instruments^1^). Samples were collected for histological analyses, fixed in FAA (3.7% formaldehyde, 5% acetic acid, 50% ethanol) under vacuum and embedded into paraffin. Sections 10 l m thick were stained in 0.2% toluidine blue solution, and observed under a Nikon Eclipse E-600 microscope^2^.

## Author Contributions

CFe conceived the project and designed the experiments together with CFo. AG-F, VS-G, CFo, and CFe performed the experiments. CFe wrote the paper.

## Conflict of Interest Statement

The authors declare that the research was conducted in the absence of any commercial or financial relationships that could be construed as a potential conflict of interest.

## Footnotes

1 http://www.oxford-instruments.com

2 http://www.nikoninstruments.com

## Supplementary Material

**FIGURE S1 | Amino acid alignment of the SHI/STY/SRS homologs included in this study.** The conserved C3HC3H RING Zinc finger and IGGH domains are highlighted in blue. In red, the atypical Pro residues in the *E. californica* proteins.

**FIGURE S2 | Sense probes for *in situ* hybridization.** **(A)** *EcSRS-L* sense probe on a transversal sections of an *E. californica* flower **(B–E)** *NbSRS-L* sense probes on transversal sections of *N. benthamiana* flowers **(B–C)** NbSRS-L1. **(D)** NbSRS-L2. **(E)** NbSRS-L3.

**FIGURE S3 | Fruits of transgenic Arabidopsis plants transformed with the 35S::EsSRS-L construct in wildtype (left)** or *sty1 sty2* **(right)** backgrounds.

**FIGURE S4 | Expression level by real-time PCR analysis of *NbSRS-L1* (orange bars), *NbSRS-L2* (pink bars) and *NbSRS-L3* (blue bars) in TRV2-NbSRS-L1, TRV2-NbSRS-L2 or TRV2-SRS-L3 flowers.** The error bars depict the s.e. based on two biological replicates. For technical problems, *NbSRS-L2* levels could not be analyzed in TRV2-SRS-L3 flowers.

**FIGURE S5 | Pistils of *E. californica* wildtype or EsSRS-L-VIGS treated plants (top panels).** Expression level by real-time PCR analysis of *EcSRS-L* in TRV2-EcSRS-L flowers. The error bars depict the s.e. based on two biological replicates.

**TABLE S1 | Protein similarity and identity matrix between Arabidopsis, *E. californica* and *N. benthamiana* SHI/STY/SRS proteins generated by MUSCLE (PAM 200).**

**TABLE S2 | Primers used in this study.**
