# Supplementary figures and images for: The Role of SHI/STY/SRS Genes in Organ Growth and Carpel Development Is Conserved in the Distant Eudicot Species Arabidopsis thaliana and Nicotiana benthamiana
Source: Front Plant Sci. 2017 May 23;8:814. doi: 10.3389/fpls.2017.00814 (PMC5440560; doi:10.3389/fpls.2017.00814)

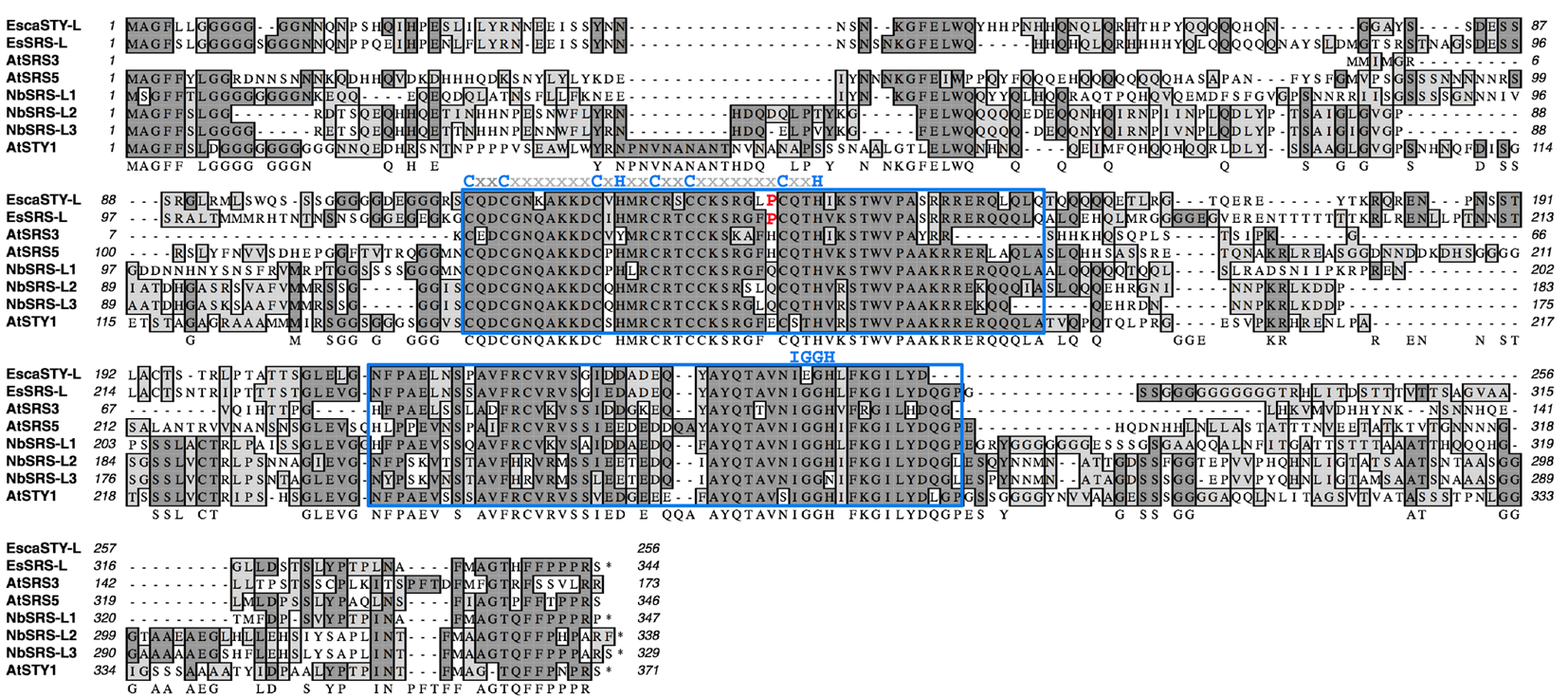

Supplement: Supplementary file 1 [file Image_1.TIF]

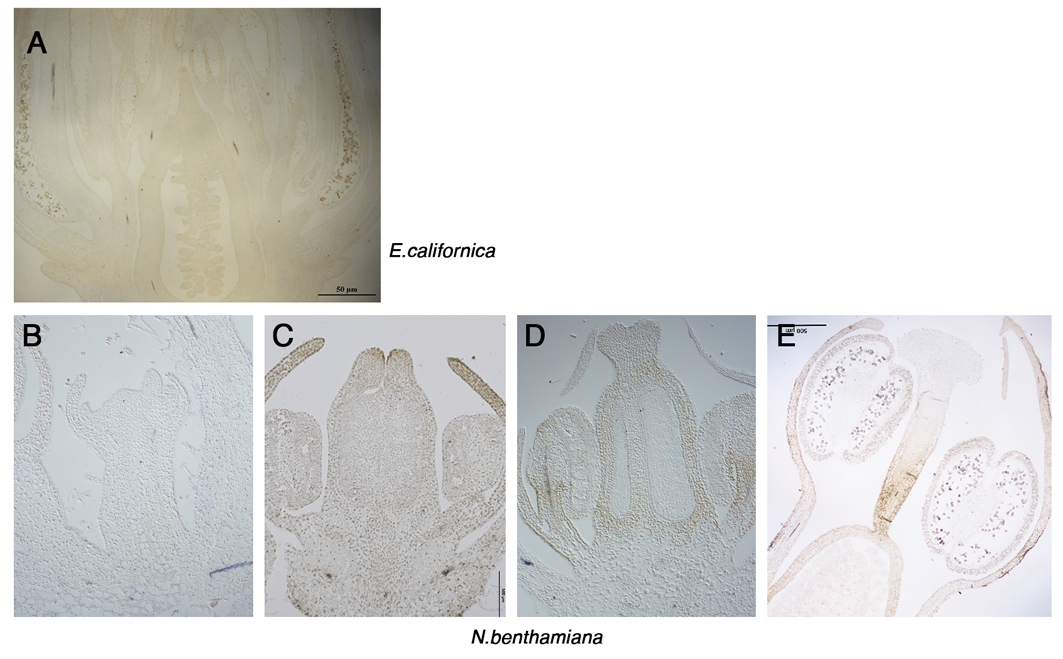

Supplement: Supplementary file 2 [file Image_2.TIF]

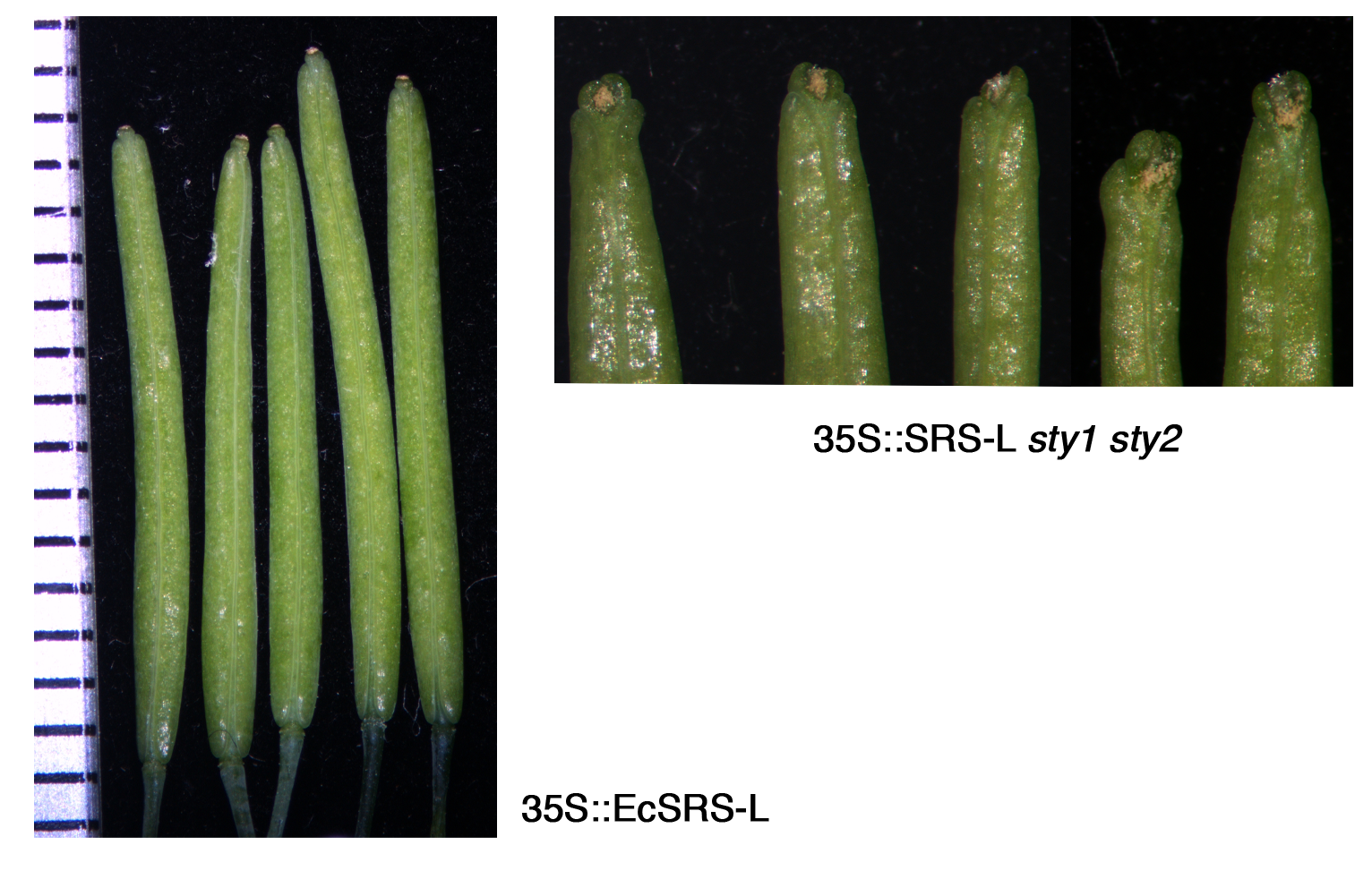

Supplement: Supplementary file 3 [file Image_3.TIF]

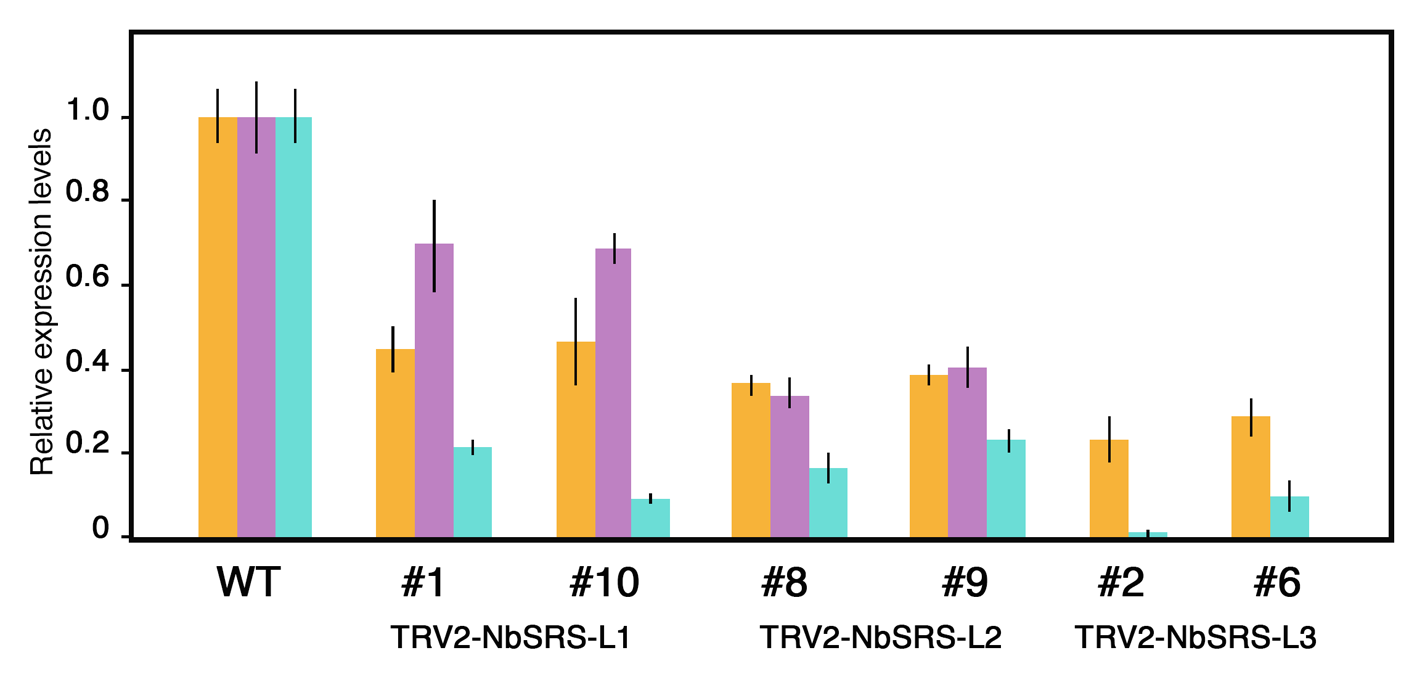

Supplement: Supplementary file 4 [file Image_4.TIF]

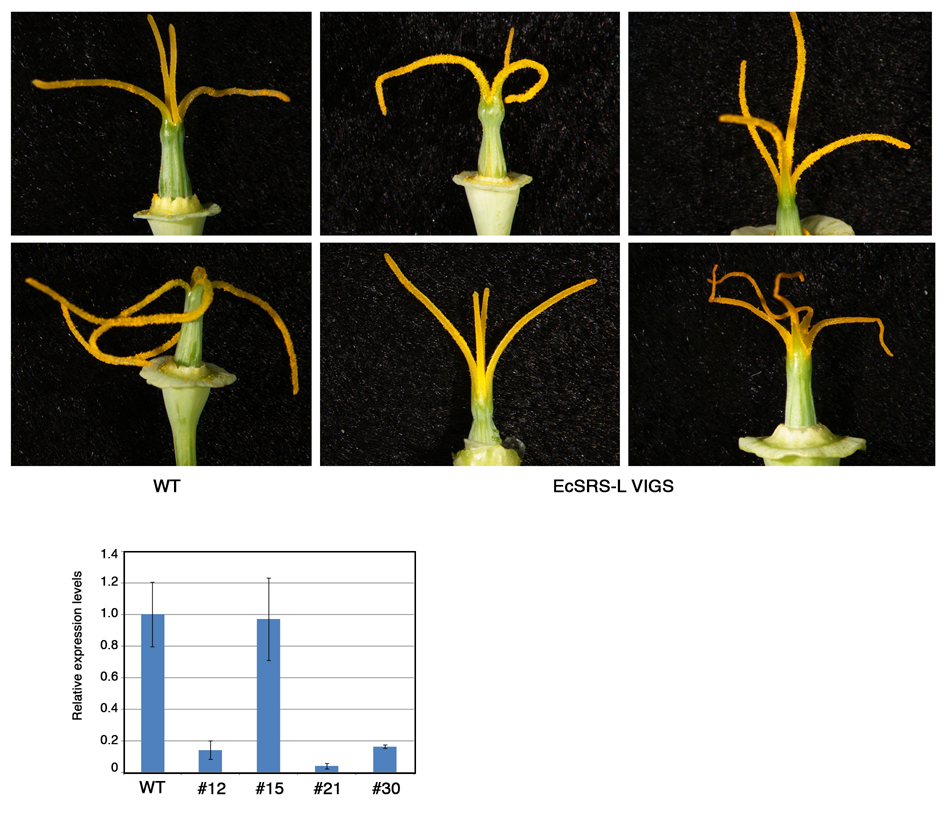

Supplement: Supplementary file 5 [file Image_5.TIF]
