# Supplementary material for: The Role of SHI/STY/SRS Genes in Organ Growth and Carpel Development Is Conserved in the Distant Eudicot Species Arabidopsis thaliana and Nicotiana benthamiana
Source: Front Plant Sci. 2017 May 23;8:814. doi: 10.3389/fpls.2017.00814 (PMC5440560; doi:10.3389/fpls.2017.00814)
Supplement: Supplementary file 6 [file Table_1.docx]

Muscle multiple sequence alignment

23 Sequences Aligned Processing time: 1.6 seconds

Gaps Inserted = 402 Conserved Identities = 16

Score = 240799

Profile Mode: PAM 200 Max Iterations: 100

Max Trees Considered: 1 Max Processing Time (hrs): 999.0

Diagonals Mode: On Anchors On

|  | **AtSTY1** | **AtSHI** | **AtSRS7** | **AtSRS5** | **AtSRS3** | **AtSRS6** | **AtSRS4** | **AtLRP1** | **AtSRS8** | **AtSRS8** | **EscaSTY** | **EsSRS-L** | **Niben101**  **Scf00197** | **Niben101**  **Scf02864** | **Niben101**  **Scf00158**  **NbSRS-L2** | **Niben101**  **Scf06228**  **NbSRS-L1** | **Niben101**  **Scf05201** | **Niben101**  **Scf00383** | **Niben101**  **Scf07034** | **Niben101**  **Scf06752** | **Niben101**  **Scf20652** | **Niben101**  **Scf21969** | **Niben101**  **Scf08273**  **NbSRS-L3** | **I**  **D**  **E**  **N**  **T**  **I**  **T**  **Y**  **S**  **C**  **O**  **R**  **E**  **(%)** |
| --- | --- | --- | --- | --- | --- | --- | --- | --- | --- | --- | --- | --- | --- | --- | --- | --- | --- | --- | --- | --- | --- | --- | --- | --- |
| **AtSTY1** | **100.0** | 42.2 | 20.9 | 23.5 | 12.2 | 12.1 | 22.5 | 14.5 | 41.9 | 23.0 | 31.7 | 27.6 | 21.8 | 28.8 | 26.8 | 26.6 | 13.3 | 28.5 | 30.0 | 11.6 | 22.7 | 24.0 | 26.1 |  |
| **AtSHI** | 50.6 | **100.0** | 32.7 | 34.0 | 21.8 | 16.0 | 26.0 | 20.6 | 21.0 | 32.3 | 32.4 | 37.0 | 31.7 | 40.4 | 37.5 | 35.5 | 18.3 | 41.3 | 43.8 | 16.3 | 34.7 | 34.7 | 37.1 |  |
| **AtSRS7** | 28.2 | 43.6 | **100.0** | 62.8 | 21.2 | 14.6 | 22.8 | 19.2 | 14.3 | 24.6 | 26.9 | 30.5 | 32.2 | 30.9 | 29.3 | 36.2 | 17.8 | 30.6 | 32.8 | 16.0 | 34.3 | 34.5 | 29.2 |  |
| **AtSRS5** | 32.2 | 49.2 | 71.5 | **100.0** | 19.9 | 16.1 | 23.2 | 18.7 | 15.1 | 26.6 | 29.7 | 36.4 | 33.6 | 34.4 | 32.8 | 39.9 | 18.1 | 34.0 | 36.2 | 16.2 | 37.5 | 37.2 | 32.8 |  |
| **AtSRS3** | 16.9 | 29.7 | 28.4 | 26.9 | **100.0** | 20.1 | 23.6 | 18.5 | 12.6 | 22.9 | 22.3 | 22.1 | 26.0 | 19.0 | 20.4 | 21.8 | 12.8 | 19.6 | 20.6 | 12.5 | 22.9 | 22.5 | 19.5 |  |
| **AtSRS6** | 18.6 | 25.7 | 23.7 | 25.5 | 32.8 | **100.0** | 18.0 | 19.9 | 12.8 | 16.4 | 19.3 | 15.9 | 18.3 | 14.9 | 16.2 | 15.9 | 13.7 | 14.4 | 15.2 | 13.2 | 18.4 | 19.0 | 16.6 |  |
| **AtSRS4** | 30.1 | 35.5 | 29.4 | 30.7 | 31.5 | 29.4 | **100.0** | 19.0 | 15.8 | 46.0 | 28.2 | 24.5 | 23.9 | 23.0 | 23.6 | 24.6 | 14.5 | 23.5 | 24.9 | 13.2 | 24.6 | 23.4 | 23.6 |  |
| **AtLRP1** | 22.3 | 31.0 | 31.0 | 30.7 | 25.1 | 28.3 | 26.0 | **100.0** | 12.8 | 21.3 | 19.0 | 21.4 | 23.0 | 20.9 | 19.9 | 20.6 | 33.9 | 20.0 | 21.3 | 32.2 | 21.7 | 21.7 | 19.7 |  |
| **AtSRS8** | 44.5 | 26.0 | 18.8 | 19.5 | 17.9 | 19.6 | 21.9 | 19.4 | **100.0** | 14.8 | 22.1 | 17.6 | 19.0 | 16.8 | 17.2 | 16.5 | 10.0 | 17.8 | 18.9 | 10.0 | 15.1 | 15.3 | 16.4 |  |
| **AtSTY2** | 31.1 | 45.1 | 35.2 | 37.5 | 30.2 | 28.4 | 54.7 | 29.7 | 19.9 | **100.0** | 27.2 | 30.7 | 28.4 | 30.8 | 29.5 | 30.6 | 17.8 | 28.7 | 30.4 | 15.5 | 31.5 | 30.5 | 29.6 |  |
| **EscaSTY** | 41.7 | 41.4 | 34.1 | 40.2 | 28.0 | 29.8 | 39.4 | 28.5 | 28.6 | 38.0 | **100.0** | **54.1** | 27.4 | 31.7 | 29.3 | 33.7 | 16.3 | 33.3 | 35.4 | 14.7 | 30.3 | 30.5 | 29.8 |  |
| **EsSRS-L** | 36.0 | 49.9 | 41.3 | 48.7 | 28.5 | 25.6 | 34.0 | 34.3 | 23.5 | 41.4 | **61.3** | **100.0** | 36.6 | 39.1 | 36.4 | 42.4 | 18.0 | 40.1 | 42.6 | 15.6 | 37.5 | 39.0 | 35.8 |  |
| **Niben101**  **Scf00197** | 27.2 | 41.2 | 41.0 | 43.7 | 35.3 | 27.3 | 31.2 | 34.3 | 24.8 | 35.9 | 33.5 | 46.6 | **100.0** | 37.4 | 34.8 | **74.9** | 18.4 | 41.2 | 43.8 | 18.4 | 43.2 | 44.6 | 34.1 |  |
| **Niben101**  **Scf02864** | 36.5 | 53.4 | 42.7 | 47.3 | 26.6 | 24.0 | 31.4 | 30.2 | 22.3 | 42.4 | 40.8 | 52.3 | 47.2 | **100.0** | 50.9 | 42.8 | 19.2 | 58.5 | 62.1 | 16.0 | 37.1 | 38.2 | 51.6 |  |
| **Niben101**  **Scf00158 NbSRS-L2** | 35.1 | 51.7 | 42.7 | 46.9 | 29.9 | 25.8 | 34.1 | 30.9 | 23.9 | 42.6 | 40.2 | 51.4 | 47.6 | 66.5 | **100.0** | 38.3 | 17.1 | 49.8 | 53.1 | 14.9 | 35.1 | 36.2 | **86.8** |  |
| **Niben101**  **Scf06228 NbSRS-L1** | 34.9 | 46.3 | 46.1 | 51.2 | 29.7 | 24.4 | 33.7 | 31.1 | 21.4 | 42.6 | 42.4 | 53.5 | **75.8** | 57.2 | 54.4 | **100.0** | 18.6 | 46.8 | 49.7 | 17.0 | 50.4 | 51.8 | 38.8 |  |
| **Niben101**  **Scf05201** | 22.5 | 28.3 | 26.3 | 29.1 | 17.4 | 20.3 | 21.9 | 42.7 | 15.5 | 27.5 | 27.1 | 30.1 | 26.7 | 29.8 | 28.3 | 28.9 | **100.0** | 17.9 | 18.5 | 53.4 | 17.3 | 18.0 | 16.5 |  |
| **Niben101**  **Scf00383** | 34.9 | 51.7 | 40.4 | 44.8 | 27.1 | 22.4 | 31.1 | 28.8 | 22.9 | 38.2 | 41.4 | 52.7 | 50.2 | 69.1 | 61.2 | 59.7 | 27.4 | **100.0** | 90.9 | 15.9 | 37.9 | 38.1 | 49.7 |  |
| **Niben101**  **Scf07034** | 36.7 | 55.1 | 42.9 | 47.4 | 28.8 | 23.8 | 33.0 | 30.1 | 24.3 | 40.5 | 44.1 | 56.1 | 53.3 | 73.3 | 65.5 | 63.4 | 28.5 | 91.7 | **100.0** | 16.5 | 40.5 | 40.8 | 53.9 |  |
| **Niben101**  **Scf06752** | 18.3 | 24.3 | 23.0 | 25.6 | 17.1 | 19.6 | 18.4 | 41.2 | 14.7 | 23.3 | 23.3 | 25.7 | 26.5 | 24.7 | 24.0 | 25.4 | 54.3 | 23.5 | 24.6 | **100.0** | 15.6 | 16.3 | 14.7 |  |
| **Niben101**  **Scf20652** | 29.6 | 46.1 | 44.1 | 50.3 | 32.9 | 28.8 | 35.0 | 31.1 | 20.2 | 42.8 | 41.4 | 51.9 | 52.3 | 49.6 | 50.7 | 60.1 | 26.9 | 49.0 | 52.3 | 23.5 | **100.0** | 91.7 | 36.7 |  |
| **Niben101**  **Scf21969** | 30.9 | 45.4 | 44.1 | 49.7 | 31.4 | 28.9 | 34.2 | 30.8 | 19.9 | 42.2 | 41.6 | 52.8 | 53.1 | 50.4 | 51.8 | 60.9 | 26.9 | 48.5 | 51.7 | 23.5 | 92.7 | **100.0** | 37.2 |  |
| **Niben101**  **Scf08273 NbSRS-L3** | 33.3 | 48.8 | 41.4 | 46.0 | 28.6 | 26.6 | 33.3 | 30.8 | 22.0 | 41.6 | 39.9 | 50.9 | 46.2 | 65.9 | **91.2** | 53.9 | 27.4 | 60.8 | 65.3 | 22.9 | 51.1 | 52.2 | **100.0** |  |
|  | **SIMILARITY SCORE (%)** | | | | | | | | | | | | | | | | | | | | | | |  |
