# Supplementary material for: The Role of SHI/STY/SRS Genes in Organ Growth and Carpel Development Is Conserved in the Distant Eudicot Species Arabidopsis thaliana and Nicotiana benthamiana
Source: Front Plant Sci. 2017 May 23;8:814. doi: 10.3389/fpls.2017.00814 (PMC5440560; doi:10.3389/fpls.2017.00814)
Supplement: Supplementary file 7 [file Table_2.DOCX]

| primer name | sequence |
| --- | --- |
| NbSTY1For | 5'- ATGTCTGGTTTTTTTACACTAG -3' |
| NbSTY1Rev | 5'- TTAAGGTCTTGGCGGTGGA -3' |
| NbSTY2For | 5'- ATGGCTGGGTTCTTTTCACTAG -3' |
| NbSTY2Rev | 5'- TCAAAATCTTGCAGGATGTG -3' |
| NbSTY3For | 5'- ATGGCTGGGTTCTTTTCACTAGG -3' |
| NbSTY3Rev | 5'- TCAAGATCTTGCAGGAGGTGG-3' |
| EcSTYF | 5'- ATGGCAGGATTTTCACTAGGTGGA-3' |
| EcSTYR | 5'- TTATGATCTTGGTGGTGGGAAG -3' |
| EcSTYdF2 | 5’-GTTGTTGCCATGGCGGCGTTG-3’ |
| EcSTYdR2 | 5’-AGATGGAAAATTCCCCACTTCTA-3’ |
| PoppyRT | 5’- GCTTGCACTTCTAATACTCGTATACCCA- 3’ |
| RT | 5’- AACTGGAAGAATTCGCGGCCGCAGGAA- 3’ |
| qNbSTY1For | 5'- AGTGGTGGGGGTATGAATTGTC -3' |
| qNbSTY1Rev | 5'- TTGGCACTGAAATCCTCTGC -3' |
| qNbSTY2For | 5'- ACCCACATACAAAGGGTTCG -3' |
| qNbSTY2Rev | 5'- CTTGGTTGCCACAATCTTGA -3' |
| qNbSTY3For | 5'- TGGTGGTGAACCAGTAGTGC -3' |
| qNbSTY3Rev | 5'- GATCTTGCAGGAGGTGGAAA -3' |
| qNbEF1For | 5'-GCACTGTGATTGATGCCCCT-3' |
| qNbEF1Rev | 5'-GGACAGCACAGTCAGCTTGG-3' |
| qEcSTYFor | 5'- CCAAAACCCACCTCAAGAAA -3' |
| qEcSTYRev | 5'- CGAACCCGCGTTAGTACTTC -3' |
| qEcACTFor | 5'-AAGAGCTCGAAACTGCCAAG-3' |
| qEcACTRev | 5'-CATCGGGAAGCTCGTAATTT-3' |
